# Does economic development contribute to sex differences in ischaemic heart disease mortality? Hong Kong as a natural experiment using a case-control study

**DOI:** 10.1186/1471-2458-8-32

**Published:** 2008-01-25

**Authors:** C Mary Schooling, Tai Hing Lam, Sai Yin Ho, Kwok Hang Mak, Gabriel M Leung

**Affiliations:** 1 Dept of Community Medicine and School of Public Health, University of Hong Kong, 21 Sassoon Road, Pokfulam, Hong Kong SAR, China; 2 Department of Health, Hong Kong SAR, China

## Abstract

**Background:**

The male excess risk of premature ischemic heart disease (IHD) mortality may be partially due to an unknown macro-environmental influence associated with economic development. We examined whether excess male risk of IHD mortality was higher with birth in an economically developed environment.

**Methods:**

We used multivariable logistic regression in a population-based case-control study of all adult deaths in Hong Kong Chinese in 1998 to compare sex differences in IHD mortality (1,189 deaths in men, 1,035 deaths in women and 20,842 controls) between Hong Kong residents born in economically developed Hong Kong or in contemporaneously undeveloped Guangdong province in China.

**Results:**

Younger (35–64 years) native-born Hong Kong men had a higher risk of IHD death than such women (odds ratio 2.91, 95% confidence interval 1.66 to 5.13), adjusted for age, socio-economic status and lifestyle. There was no such sex difference in Hong Kong residents who had migrated from Guangdong. There were no sex differences in pneumonia deaths by birth place.

**Conclusion:**

Most of these people migrated as young adults; we speculate that environmentally mediated differences in pubertal maturation (when the male disadvantage in lipids and fat patterning emerges) may contribute to excess male premature IHD mortality in developed environments.

## Background

At older ages women are as vulnerable to heart disease as men. However, men are more vulnerable to premature mortality from ischemic heart disease (IHD) [1,2]. Estrogens undoubtedly play a part, but geographic differences [2], the lack of inflection in coronary heart disease mortality at menopause [3], and the essentially null results of hormone replacement therapy trials [4] suggest estrogens may not be the only factor [2]. The excess IHD risk in men is currently smallest in economically undeveloped locations, such as rural China [2], whilst it increased during the first half of the 20^th ^century in some industrialised countries [1,2]. These geo-temporal differences suggest that some as yet unidentified environmental factors [1] possibly associated with economic development may be relevant. Migration from less developed to more economically developed countries provide a parallel with socio-economic development. Migration studies, such as the Honolulu Heart Study have been instrumental in understanding the trends in and environmental determinants of IHD [5]. Migration to more economically developed countries is usually associated with increased IHD risk, although, migration studies have not provided consistently converging scientific viewpoints [6]. The many different factors involved, i.e. ethnicity, country of origin and destination, acculturation, age at migration and generations since migration may all contribute to these disparate interpretations [7-11]. To our knowledge, no previous study has examined sex differences in IHD mortality with migration to a more economically developed location within an ethnically homogenous population. We took advantage of a natural experiment provided by migration into Hong Kong and a large population-based case-control study to examine if male excess risk of IHD mortality was associated with living in an economically developed location.

Most Chinese people in Hong Kong are first or second generation migrants from the neighbouring province of Guangdong in southern China. The main waves of migration took place in the late 1940s and early 1950s [12]. Industrialization and economic growth started in the first half of the 20^th ^century in Hong Kong [13], by 1952 per capita gross domestic product (GDP) was half that of western Europe and by 1995 had overtaken western Europe [14]. In contrast, China had a GDP per head similar to pre-industrialized western Europe until the 1970s [15]. Economic development in Guangdong province was not a priority until the establishment of the Special Economic Zones near Hong Kong in 1978, and despite substantial economic growth thereafter Guangdong GDP per head in US dollars was less than a tenth of levels in Hong Kong in 2001 [16]. The population of Hong Kong is ethnically (>95% Chinese), culturally and most likely genetically homogeneous, with the main difference between migrants and non-migrants being the duration and number of generations of economic development experienced, making Hong Kong unlike many other seminal migration studies [17], whilst fortuitously uniquely suited to examining the impact of economic development alone. Hong Kong also has a low prevalence of IHD compared with western countries or other developed Chinese communities, such as Singapore [18], whose population has a longer history of economic development. We hypothesised that if male excess risk of premature IHD mortality is driven by economic development and possibly also the associated nutritional transformation, we should observe a larger male excess risk of IHD mortality in people born and brought up in Hong Kong than in people who migrated to Hong Kong from China Guangdong province mainly as young adults [19]. To allow for the possibility of "salmon bias" [20] (i.e. unhealthy migrants returning home to die), or "healthy migrant" bias (i.e. self-selection of healthy people as migrants) disproportionately affecting male 'breadwinners' we also examined sex differences in risk of mortality from a cause where no differences would be expected, i.e. pneumonia. We used pneumonia in preference to all respiratory deaths to avoid changing patterns of tuberculosis and chronic obstructive pulmonary disease. In the present study we examined whether the male excess risk of premature mortality from IHD, but not pneumonia, was higher with birth in an economically developed environment.

## Method

### Sources of data

The LIMOR (LIfestyle and MORtality) study is a population-based case-control study of adult deaths among ethnic Chinese residents in Hong Kong during mid-December 1997 to mid-January 1999. The study was originally designed to examine the effect of smoking on mortality [21]. It captured 81% of all deaths registered in Hong Kong during the study period, with the cause of death routinely coded by the Governmental Department of Health. Almost all deaths in Hong Kong are certified by hospital doctors and validity and completeness of the causes of death should be good; Hong Kong cause of death coding has been used in other studies and found to be of good quality [21]. Using a standardised questionnaire, information on current age, sex, place of birth and educational attainment and health behaviours (smoking, use of alcohol, dietary habits and leisure exercise), employment and housing ten years previously of the decedent and a similar living person aged 60 years or older was collected from the person registering the death, who was usually one of the more educated adult children of the deceased's family, often living in close, multi-generational Chinese families. Unfortunately, the large-scale population movements in the mid 20^th ^century in this region and the relatively recent economic development preclude ideal data sources such as pre-existing long-term, population-representative cohort studies. Nevertheless, proxy informants can provide adequate information [22], and we have used this study previously to examine the impact on mortality of physical activity, second-hand smoke exposure and diet [23-25].

### Birthplace

Place of birth was recorded as Hong Kong, Guangdong province of China, other parts of China, Macau and elsewhere. To increase ethnic homogeneity and to reduce confounding by other unmeasured factors we only included people born in Hong Kong or neighboring Guangdong province (88.1% of all deaths).

### Statistical analysis

The major outcomes were cause-specific mortality from IHD (ICD-9: 410–414) and pneumonia (ICD-9: 485–6). We exploited the comprehensive, population-based nature of the decedents in this sample to examine whether there was the same sex ratio in IHD and pneumonia deaths for native born Hong Kong residents and Guangdong born Hong Kong residents, using other deaths as controls. This design enables a direct comparison of men and women, which is not appropriate using living controls as there are more female than male living controls, and relatively few young controls. In a case-control mortality study the odds ratio obtained using other deaths as controls produces an unbiased estimator of the mortality rate ratio (or relative risk) if the exposure in question has no effect on relative risk in the controls [26]. Our hypothesis concerns the sex ratio in IHD mortality for people with different experiences of economic development. How the sex-ratio in mortality from other diseases changes with economic development is unclear. We used two sets of controls; first, all other deaths and second all other deaths excluding hormone related cancers, i.e. breast cancer (ICD-9: 174), prostate cancer (ICD-9: 185), ovarian cancer (ICD-9: 183), and endometrial cancer (ICD-9: 182), and also diabetes (ICD-9: 250), because hormone related cancers might become more common in women than men with economic development, whilst diabetes appears to become less common [27]. We classed death from IHD before 65 years as premature and so considered two age groups, 35–<65 years and 65 years or above.

Unconditional logistic regression adjusted for potential confounders (collected in the original survey) was used to assess the association of sex with IHD mortality by place of birth. Potential confounders considered were age (in 5 year age-groups), education (no formal, primary or secondary), housing type (hut/shared, public estate, self-owned or other), smoking status (never, ex-smoker or current smoker), alcohol use (ever or never), leisure exercise (less than once a month or at least once a month) and frequency of consumption of tea, dairy products, fish, meat, vegetables, fruit and soy (less than 4 times a week or at least 4 times a week). However, after preliminary analysis dietary items were not included because they did not change the effect sizes for IHD by more than 5%. Moreover, as some of the remaining confounders, such as smoking, could potentially be on the casual pathway we presented two models; model 1 adjusted only for age and model 2 additionally adjusted for education, housing type, smoking, leisure exercise and alcohol use. We assessed whether the sex ratio differed with birthplace from the heterogeneity of effect across strata and the model fit, (i.e. whether a model with an interaction term for birthplace with sex had a smaller Akaike Information Criteria (AIC)). Complete data were available for 95% of the relevant observations; missing data were excluded. The project received ethics approval from the Ethics Committee of the Faculty of Medicine, The University of Hong Kong.

## Results

Consistent with all deaths in Hong Kong in 1998,[28] the leading cause of death in the study was cancer (36%), followed by diseases of the circulatory system (27%) and respiratory system (20%). Of the 27,444 deaths aged over 35 years in the original study, 3262 were excluded because of birth outside Hong Kong or Guangdong province and 1116 had some missing data leaving 23,066 deaths. There were 1,189 and 1,035 deaths from IHD in men and women respectively and 1,322 and 1,387 from pneumonia. There were 20,842 deaths from causes other than IHD, 19,945 deaths from causes other than IHD, prostate cancer, breast cancer, uterine cancer and diabetes and 20357 deaths from causes other than pneumonia. Table 1 shows the characteristics of the IHD and pneumonia cases and their respective controls. Almost all the participants (98.5%) had lived in Hong Kong for more than 20 years. As is typical of the region, and specifically Hong Kong, men were much better educated than women [29].

**Table 1:** Characteristics of cases and controls for Chinese men and women from Hong Kong in 1998

		35–64					65+				
		IHD			Pneumonia		IHD			Pneumonia	
		
Characteristic		Cases	Controls †	Restricted controls ‡	Cases	Controls §	Cases	Controls †	Restricted controls ‡	Cases	Controls §
Men	N	224	3190	3146	127	3287	965	8382	8153	1195	8152
Age	Mean	56.7	53.7	53.7	55.2	53.9	76.9	76.8	76.7	80.9	76.2
Education	No formal (%)	10	12	12	17	11	28	33	33	36	32
	Primary (%)	52	48	48	55	48	49	48	48	45	49
	Secondary (%)	38	40	40	28	41	23	19	19	19	19
Housing type	Hut/shared (%)	11	13	13	12	13	7	10	10	9	9
	Public estate (%)	47	49	49	51	48	45	49	49	48	49
	Self-owned (%)	38	33	33	27	33	42	35	35	34	36
	Other (%)	4	5	5	10	5	5	7	7	9	6
Birthplace	Hong Kong (%)	38	38	38	35	39	15	12	12	12	13
*10 years previously*											
Exercise	<1/month (%)	76	75	75	86	74	64	69	69	69	68
Smoking	Never (%)	35	33	33	23	34	36	28	28	34	28
	Ex-smoker (%)	7	6	6	8	6	20	21	21	24	21
	Current smoker (%)	58	61	61	69	60	44	51	51	43	51
Alcohol use	Never (%)	47	56	44	45	43	57	48	48	54	48
Women	N	68	1553	1314	48	1573	967	7717	7332	1339	7345
Age	Mean	57.5	53.0	53.3	54.1	53.1	81.0	80.9	81.1	85.6	80.0
Education	No formal (%)	35	24	26	38	24	70	71	71	75	70
	Primary (%)	40	45	45	42	45	22	23	23	18	24
	Secondary (%)	25	31	29	21	31	8	6	6	7	6
Housing type	Hut/shared (%)	7	8	8	13	8	9	9	9	10	8
	Public estate (%)	49	50	51	54	50	46	45	46	43	46
	Self-owned (%)	38	38	37	29	38	39	37	37	33	38
	Other (%)	6	4	4	4	4	6	9	9	14	7
Birthplace	Hong Kong (%)	29	45	44	38	45	13	13	14	13	13
*10 years previously*											
Exercise	<1/month (%)	74	69	69	77	69	63	66	66	71	64
Smoking	Never (%)	88	90	89	85	90	79	76	76	80	76
	Ex-smoker (%)	1	1	1	0	1	7	9	9	8	8
	Current smoker (%)	10	9	10	14	9	14	15	15	12	15
Alcohol use	Never (%)	87	89	89	85	89	89	87	87	87	87

IHD = ischaemic heart disease

† all deaths excluding IHD deaths

‡ all deaths excluding deaths from IHD and hormone related cancers, i.e. breast cancer (ICD-9: 174), prostate cancer (ICD-9: 185), ovarian cancer (ICD-9: 183), and endometrial cancer (ICD-9: 182), and also diabetes (ICD-9: 250)

§ all deaths excluding deaths from pneumonia

**Figure 1:**
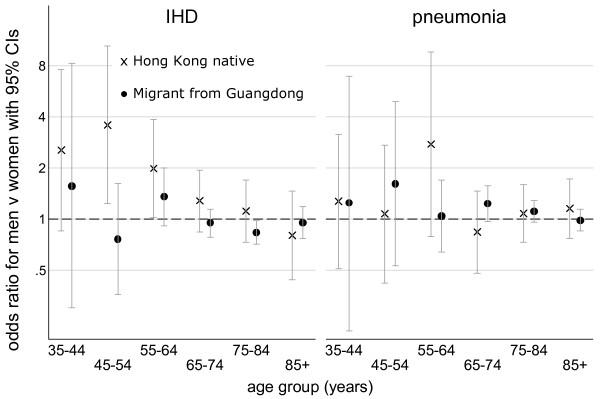
Unadjusted odds ratio of IHD and pneumonia mortality in men compared with women stratified by place of birth and 10 year age-group.

Figure 1 shows the unadjusted odds ratios of dying from IHD or pneumonia in men compared with women stratified by birth-place and 10 year age-group, i.e. the reference group is women. In the younger age-groups (35–44, 45–54 and 55–64 years) Hong Kong born men were about twice as likely to die of IHD than Hong Kong born women, however such differences were less clear for the Guangdong born or for deaths from pneumonia. Similarly Table 2 shows that younger (35 to 64 years) Hong Kong born men were significantly more likely to die from IHD than similar Hong Kong born women using either set of controls, adjusted for age (model 1) and also additionally adjusted for socio-economic status and lifestyle (model 2). However, for Hong Kong residents born in Guangdong men and women were equally likely to die from IHD. Including a sex with birth-place interaction term yielded a lower AIC in both models with either set of controls. In the older people (65 years and older) the difference between the sexes in dying from IHD was much less evident with all odds ratios fairly close to 1, although the AIC was slightly lower in a model with a sex with birth-place interaction term. In contrast, men were slightly more likely than women to die from pneumonia in both age-groups regardless of place of birth, which is consistent with the shorter life expectancy in men more generally.

**Table 2:** Adjusted odds ratio of IHD and pneumonia mortality in men compared with women (reference group OR = 1) stratified by place of birth and age-group

Cause	Age group	Place of birth	All other deaths as controls				Restricted† deaths as controls			
			Model 1		Model 2		Model 1		Model 2	
	(years)		OR	95% CI	OR	95% CI	OR	95% CI	OR	95% CI
IHD	35–64	Hong Kong native	2.44	1.48 to 4.02	2.91	1.66 to 5.13	2.08	1.26 to 3.44	2.46	1.39 to 4.34
		Migrant from Guangdong	1.23	0.87 to 1.72	1.35	0.89 to 2.06	1.10	0.78 to 1.54	1.22	0.80 to 1.85
	65+	Hong Kong native	1.11	0.85 to 1.44	1.27	0.72 to 1.40	1.08	0.83 to 1.40	1.24	0.91 to 1.70
		Migrant from Guangdong	0.90	0.81 to 1.00	1.03	0.90 to 1.17	0.87	0.78 to 0.97	1.00	0.88 to 1.14
Pneumonia	35–64	Hong Kong native	1.37	0.79 to 2.39	1.19	0.60 to 2.34				
		Migrant from Guangdong	1.18	0.77 to 1.80	1.07	0.61 to 1.85				
	65+	Hong Kong native	1.17	0.90 to 1.50	1.16	0.85 to 1.59				
		Migrant from Guangdong	1.15	1.04 to 1.26	1.22	1.09 to 1.38				

Model 1 adjusted for age (5 year groups)

Model 2 adjusted for age (5 year groups), education (no formal, primary or secondary), housing type (hut/shared, public estate, self-owned or other), smoking (never, ex-smoker or current smoker), leisure exercise (less than once a month or at least once a month) and alcohol use (ever or never)

†restricted controls are all deaths, apart from deaths from IHD and cancers of the breast, prostate, ovary and uterus

Table 3 shows the odds ratio of IHD or pneumonia mortality in Hong Kong born natives compared with migrants from Guangdong province stratified by sex and age group and adjusted for age (model 1) and additionally adjusted for socio-economic status and lifestyle (model 2). Younger Hong Kong born men were more likely to die from IHD than similar Guangdong born men, but Hong Kong born women were no more likely to die from IHD than Guangdong born women, these differences were smaller for IHD mortality in older men and women. Pneumonia mortality was similar regardless of place of birth.

**Table 3:** Adjusted odds ratio of IHD and pneumonia mortality in Hong Kong natives compared with migrants from Guangdong by sex and age-group

Cause	Age group	Sex	Migration status	All other deaths as controls				Restricted† deaths as controls			
				Model 1		Model 2		Model 1		Model 2	
	(years)			OR	95% CI	OR	95% CI	OR	95% CI	OR	95% CI

IHD	35–64	Men	Migrant from Guangdong	1		1		1		1	
			Hong Kong native	1.39	1.03 to 1.88	1.35	1.00 to 1.84	1.40	1.04 to 1.90	1.36	1.00 to 1.85
		Women	Migrant from Guangdong	1		1		1		1	
			Hong Kong native	0.71	0.40 to 1.24	0.68	0.38 to 1.21	0.73	0.41 to 1.29	0.70	0.40 to 1.26
	65+	Men	Migrant from Guangdong	1		1		1		1	
			Hong Kong native	1.24	1.03 to 1.50	1.19	0.98 to 1.45	1.24	1.03 to 1.50	1.20	0.99 to 1.45
		Women	Migrant from Guangdong	1		1		1		1	
			Hong Kong native	0.97	0.80 to 1.19	0.97	0.80 to 1.19	0.96	0.79 to 1.18	0.96	0.79 to 1.18
Pneumonia	35–64	Men	Migrant from Guangdong	1		1					
			Hong Kong native	0.94	0.63 to 1.42	1.00	0.66 to 1.52				
		Women	Migrant from Guangdong	1		1					
			Hong Kong native	0.76	0.40 to 1.46	0.83	0.43 to 1.60				
	65+	Men	Migrant from Guangdong	1		1					
			Hong Kong native	1.00	0.82 to 1.21	1.00	0.82 to 1.20				
		Women	Migrant from Guangdong	1		1					
			Hong Kong native	1.01	0.84 to 1.21	0.96	0.80 to 1.15				

Model 1 adjusted for age (5 year groups)

Model 2 adjusted for age (5 year groups), education (no formal, primary or secondary), housing type (hut/shared, public estate, self-owned or other), smoking (never, ex-smoker or current smoker), leisure exercise (less than once a month or at least once a month) and alcohol use (ever or never)

† restricted controls are all other deaths, apart from deaths from IHD and cancers of the breast, prostate, ovary and uterus.

## Discussion

Taking advantage of a unique natural experiment, we found male excess risk of premature IHD mortality was related to environmental factors. Men, who were born and brought up in the economically developed environment of Hong Kong, had an excess risk of premature IHD mortality compared to women, but there was no such difference for Hong Kong residents who had migrated from Guangdong. On the other hand at older ages men and women had more similar risks of dying from IHD regardless of place of birth. To our knowledge, these findings are unique. They confirm our hypothesis that consistent with findings from ecological studies [1,2] a lifetime spent in an economically developed environment increases the risk of premature mortality from IHD in men but not women. Given, that most migration into Hong Kong took place in the late 1940s and early 1950s our findings also suggest that the relevant environmental influence is in the first two or three decades of life.

Nevertheless, factors other than environmental exposures across the life course may be relevant. First, unhealthy migrant men from Guangdong may have preferentially returned home, whilst such women did not. However, political differences between China and the former colonial power, the provision of Hong Kong residency to migrants, the much higher living standards in Hong Kong and the universal access to health care, education and social welfare in Hong Kong make large-scale migration back to China prior to 1998 is extremely unlikely. Alternatively, men from Guangdong may have migrated as self-selected healthy workers, whilst women migrated as unselected family members, although why a healthy migrant effect should be specific to IHD is difficult to explain. Due to historical circumstances, there was no census in Hong Kong between 1931 and 1961 and very little control on migration into Hong Kong during that period. The only source we have been able to identify which describes the main post-war influx of migrants into Hong Kong describes them as mainly young people looking for work, however the basis for that observation is impressionistic [19]. Second, in adult life men and women may respond differently to living in an economically developed region depending on their childhood location. Possibly men, but not women, from Guangdong continued a protective 'Guangdong' lifestyle, whilst men, but not women, of Hong Kong had a more 'westernized' lifestyle with its corresponding risks. Alternatively, men and women may have responded differently to the stresses of migration and living in Hong Kong, with corresponding increases in IHD risk [30]. Although, stress has similar effects on IHD risk in men and women [30], it is possible that life in Hong Kong was particularly stressful for migrant women and Hong Kong born men. There could also be differential residual confounding in men and women. However, if either of these were the case there should also have been differences in pneumonia mortality by sex and place of birth, which were not observed. Third, case-control studies are subject to recall bias, however simple information appropriate to proxy recall was collected [31,32]. Moreover, cause of death was obtained from a well-maintained registry. There is universal access to health care in Hong Kong and most Hong Kong residents die in modern hospitals providing western medical care, so ascertainment of cause of death cause of is generally very good. However, inevitably some deaths may be misclassified, which would make our results conservative. Finally, using decedent controls in a mortality case control study would under-estimate male excess IHD risk if exposure to an economically developed environment preferentially increased risk of death from non-IHD causes in men, and would conversely over-estimate male excess IHD risk if the same exposure preferentially increased risk of death from non-IHD causes in women. However, we obtained similar results after removing from the controls deaths from causes which might have a sex-specific relationship with economic development, such as diabetes or cancer of the breast, prostate, uterus or ovary.

Given that we took advantage of a natural experiment, we are not showing precise point estimates of differences by sex with economic development, although they are broadly consistent with estimates in populations at different stages of economic development [2]. In particular, our estimates may be conservative. Age at migration and duration of residence in Hong Kong were not collected in the study. Some people born in Guangdong undoubtedly migrated to Hong Kong as children or adolescents. On the other hand some Hong Kong born people may have grown up elsewhere and returned later to Hong Kong. Men and women probably had essentially similar and comparable experiences, but the exposures to different livings conditions were diluted in some people, making our results conservative. In addition economic differentials between Hong Kong and Guangdong province were not consistent across the 20^th ^century, and specifically may have been smaller in the early 20^th ^century when the older people were growing up. Thus, it is possible that the lack of sex difference in IHD mortality at older ages may be partially artifactual due to smaller differences in living conditions between Hong Kong and Guangdong in the early 20^th ^century. Nevertheless, our study provides aetiologically significant evidence that male excess risk of premature IHD mortality is partially generated by a macro-environmental influence associated with economic development operating in the first few decades of life, which might increase the risk of IHD in men, decrease the risk of IHD in women or increase the risk of death from other causes in women.

Notwithstanding these provisos our study suggests that a factor in the first few decades of life contributes to the higher male risk of premature IHD mortality that emerges with economic development. Several aspects of pre-adult life are associated with increased cardiovascular risk, such as accelerated growth [33,34], shorter legs and lower leg to trunk ratios [35,36]. There is little indication that these factors have different associations by sex. On the other hand, puberty is the specific period of life when sex differences become much more pronounced due to the action of testosterone in males and estrogen in females. Increased testosterone in males at puberty also increases some IHD risk factors, such as reduced HDL-cholesterol [37-39], and possibly higher ApoB and more male fat patterning [39]. Conversely, higher estrogen levels at puberty promote a more female shape [40] with a lower waist-hip-ratio and corresponding lower IHD risk, but possibly also a higher risk of some hormone related cancers [41,42]. Moreover, sex-differences in HDL-cholesterol appear to be contextually specific and smallest in least developed locations, such as China [43], suggesting they are environmentally driven, although these findings have not always been replicated in small studies [44].

In animal experiments under-feeding reduces testosterone [45-47] and estrogen levels [48] at puberty. Even in universally well-fed human populations, slight changes in diet affect sex-steroids in girls [49] although not in boys [50], where larger nutritional differences are needed [51]. We speculate that nutritionally driven differences in pubertal development seen in developed environments [52] but not very poor environments [51], may permanently increase sex differences in IHD risk, through higher levels of sex-steroids at puberty generating more detrimental lipids and body shape in men, whilst generating a more female body shape in women. Such differences would be expected to track into adult life [53]. Moreover, consistent with the much less marked change with economic development in the sex ratio for diabetes [27] than for IHD, such environmentally driven effects on pubertal sex-steroids would not be expected to affect pubertal pre-cursors of diabetes, which appear to be more strongly related to another hypothalamic-pituitary-endocrine axis, i.e. growth hormone [54-59].

## Conclusion

Rather than being entirely an intrinsic sex-specific property, increased male risk of premature IHD mortality was associated with living in an economically developed environment in the first few decades of life. We speculate that a better pre-adult environment increases sex-steriods at puberty, which permanently increases IHD mortality risk in men whilst possibly decreasing IHD risk in women but possibly also increasing risks of other diseases, thus providing a socio-biological explanation for the emergence of increased male risk of premature IHD mortality following economic development. Our findings highlight the importance of investigating the long-term effects of early life environment on cardiovascular risk in the large proportion of the global population under-going rapid epidemiologic transition.

## Competing interests

The author(s) declare that they have no competing interests.

## Authors' contributions

CMS and GML designed this analysis and drafted the manuscript. THL, SYH and KHM designed the original study and acquired the data. All authors revised the manuscript critically for important intellectual content; and gave final approval of the version to be published.

## Pre-publication history

The pre-publication history for this paper can be accessed here:



## References

[B1] Nikiforov SV, Mamaev VB (1998). The development of sex differences in cardiovascular disease mortality: a historical perspective. Am J Public Health.

[B2] Lawlor DA, Ebrahim S, Smith GD (2001). Sex matters: secular and geographical trends in sex differences in coronary heart disease mortality. BMJ.

[B3] Lawlor DA, Ebrahim S, Smith GD (2002). Role of endogenous oestrogen in aetiology of coronary heart disease: analysis of age related trends in coronary heart disease and breast cancer in England and Wales and Japan. BMJ.

[B4] Cho L, Mukherjee D (2005). Hormone replacement therapy and secondary cardiovascular prevention: a meta-analysis of randomized trials. Cardiology.

[B5] Benfante R (1992). Studies of cardiovascular disease and cause-specific mortality trends in Japanese-American men living in Hawaii and risk factor comparisons with other Japanese populations in the Pacific region: a review. Hum Biol.

[B6] Misra A, Ganda OP (2007). Migration and its impact on adiposity and type 2 diabetes. Nutrition.

[B7] McKeigue PM, Miller GJ, Marmot MG (1989). Coronary heart disease in south Asians overseas: a review. J Clin Epidemiol.

[B8] Razum O, Zeeb H, Gerhardus A (1998). Cardiovascular mortality of Turkish nationals residing in West Germany. Ann Epidemiol.

[B9] Freire RD, Cardoso MA, Shinzato AR, Ferreira SR (2003). Nutritional status of Japanese-Brazilian subjects: comparison across gender and generation. Br J Nutr.

[B10] Sundquist K, Li X (2006). Coronary heart disease risks in first- and second-generation immigrants in Sweden: a follow-up study. J Intern Med.

[B11] Schooling M, Leung GM, Janus ED, Ho SY, Hedley AJ, Lam TH (2004). Childhood migration and cardiovascular risk. Int J Epidemiol.

[B12] Tsang S (2004). A Modern History of Hong Kong.

[B13] Ngo TW, Faure D (2003). Industrial History and the Artiface of Laissez-faire Colonialism. Hong Kong: A Reader in Social History.

[B14] Dahlman CJ, Aubert JE (2001). China and the knowledge economy seizing the 21st century.

[B15] Maddison A (1998). Chinese economic performance in the long run.

[B16] Richard WYC (2002). Shanghai: Another Hong Kong. HKCER Letters.

[B17] Elford J, Ben-Shlomo Y, Kuh D and Ben Shlomo Y (2004). Geography and migration with special reference to cardiovascular disease. A life course approach to chronic disease epidemiology.

[B18] Dwyer T, Emmanuel SC, Janus ED, Wu Z, Hynes KL, Zhang C (2003). The emergence of coronary heart disease in populations of Chinese descent. Atherosclerosis.

[B19] (1955). Hong Kong Annual Report-1954.

[B20] Abraido-Lanza AF, Dohrenwend BP, Ng-Mak DS, Turner JB (1999). The Latino mortality paradox: a test of the "salmon bias" and healthy migrant hypotheses. Am J Public Health.

[B21] Lam TH, Ho SY, Hedley AJ, Mak KH, Peto R (2001). Mortality and smoking in Hong Kong: case-control study of all adult deaths in 1998. BMJ.

[B22] Campbell PT, Sloan M, Kreiger N (2007). Utility of proxy versus index respondent information in a population-based case-control study of rapidly fatal cancers. Ann Epidemiol.

[B23] Lam TH, Ho SY, Hedley AJ, Mak KH, Leung GM (2004). Leisure time physical activity and mortality in hong kong: case-control study of all adult deaths in 1998. Ann Epidemiol.

[B24] McGhee SM, Ho SY, Schooling M, Ho LM, Thomas GN, Hedley AJ, Mak KH, Peto R, Lam TH (2005). Mortality associated with passive smoking in Hong Kong. BMJ.

[B25] Schooling CM, Ho SY, Leung GM, Thomas GN, McGhee SM, Mak KH, Lam TH (2006). Diet synergies and mortality--a population-based case-control study of 32,462 Hong Kong Chinese older adults. Int J Epidemiol.

[B26] Calle EE (1984). Criteria for selection of decedent versus living controls in a mortality case-control study. Am J Epidemiol.

[B27] Gale EA, Gillespie KM (2001). Diabetes and gender. Diabetologia.

[B28] Chan M (1999). Annual Departmental Report for the Financial Year 1998-1999.

[B29] Schooling CM, Thomas GN, Leung GM, Ho SY, Janus ED, Lam TH (2007). Is height associated with cardiovascular risk in Chinese?. Epidemiology.

[B30] Rosengren A, Hawken S, Ounpuu S, Sliwa K, Zubaid M, Almahmeed WA, Blackett KN, Sitthi-amorn C, Sato H, Yusuf S (2004). Association of psychosocial risk factors with risk of acute myocardial infarction in 11119 cases and 13648 controls from 52 countries (the INTERHEART study): case-control study. Lancet.

[B31] Herrmann N (1985). Retrospective information from questionnaires. I. Comparability of primary respondents and their next-of-kin. Am J Epidemiol.

[B32] Lerchen ML, Samet JM (1986). An assessment of the validity of questionnaire responses provided by a surviving spouse. Am J Epidemiol.

[B33] Eriksson JG, Forsen T, Tuomilehto J, Winter PD, Osmond C, Barker DJ (1999). Catch-up growth in childhood and death from coronary heart disease: longitudinal study. BMJ.

[B34] Singhal A, Cole TJ, Fewtrell M, Deanfield J, Lucas A (2004). Is slower early growth beneficial for long-term cardiovascular health?. Circulation.

[B35] Gunnell DJ, Smith GD, Frankel S, Nanchahal K, Braddon FE, Pemberton J, Peters TJ (1998). Childhood leg length and adult mortality: follow up of the Carnegie (Boyd Orr) Survey of Diet and Health in Pre-war Britain. J Epidemiol Community Health.

[B36] Smith GD, Greenwood R, Gunnell D, Sweetnam P, Yarnell J, Elwood P (2001). Leg length, insulin resistance, and coronary heart disease risk: the Caerphilly Study. J Epidemiol Community Health.

[B37] Kirkland RT, Keenan BS, Probstfield JL, Patsch W, Lin TL, Clayton GW, Insull W (1987). Decrease in plasma high-density lipoprotein cholesterol levels at puberty in boys with delayed adolescence. Correlation with plasma testosterone levels. JAMA.

[B38] Hinkel GK, Hanefeld M, Jaross W, Leonhardt W, Trubsbach A (1985). Effects of high doses of oestrogens and androgens on lipoproteins: observations in the treatment of excessive growth with sexual hormones. Exp Clin Endocrinol.

[B39] Morrison JA, Barton BA, Biro FM, Sprecher DL (2003). Sex hormones and the changes in adolescent male lipids: longitudinal studies in a biracial cohort. J Pediatr.

[B40] Roemmich JN, Rogol AD (1999). Hormonal changes during puberty and their relationship to fat distribution. Am J Human Biol.

[B41] Hamilton AS, Mack TM (2003). Puberty and genetic susceptibility to breast cancer in a case-control study in twins. N Engl J Med.

[B42] De Assis S, Hilakivi-Clarke L (2006). Timing of dietary estrogenic exposures and breast cancer risk. Ann N Y Acad Sci.

[B43] Davis CE, Williams DH, Oganov RG, Tao SC, Rywik SL, Stein Y, Little JA (1996). Sex difference in high density lipoprotein cholesterol in six countries. Am J Epidemiol.

[B44] Lindeberg S, Ahren B, Nilsson A, Cordain L, Nilsson-Ehle P, Vessby B (2003). Determinants of serum triglycerides and high-density lipoprotein cholesterol in traditional Trobriand Islanders: the Kitava Study. Scand J Clin Lab Invest.

[B45] Adam CL, Findlay PA (1997). Effect of nutrition on testicular growth and plasma concentrations of gonadotrophins, testosterone and insulin-like growth factor I (IGF-I) in pubertal male Soay sheep. J Reprod Fertil.

[B46] Nolan CJ, Neuendorff DA, Godfrey RW, Harms PG, Welsh TH, McArthur NH, Randel RD (1990). Influence of dietary energy intake on prepubertal development of Brahman bulls. J Anim Sci.

[B47] Slob AK, Vreeburg JT, Van der Werff ten Bosch JJ (1979). Body growth, puberty and undernutrition in the male guinea-pig. Br J Nutr.

[B48] Ronnekleiv OK, Ojeda SR, McCann SM (1978). Undernutrition, puberty and the development of estrogen positive feedback in the female rat. Biol Reprod.

[B49] Dorgan JF, Hunsberger SA, McMahon RP, Kwiterovich PO, Lauer RM, Van Horn L, Lasser NL, Stevens VJ, Friedman LA, Yanovski JA, Greenhut SF, Chandler DW, Franklin FA, Barton BA, Buckman DW, Snetselaar LG, Patterson BH, Schatzkin A, Taylor PR (2003). Diet and sex hormones in girls: findings from a randomized controlled clinical trial. J Natl Cancer Inst.

[B50] Dorgan JF, McMahon RP, Friedman LA, Van Horn L, Snetselaar LG, Kwiterovich PO, Lauer RM, Lasser NL, Stevens VJ, Robson A, Cooper SF, Chandler DW, Franklin FA, Barton BA, Patterson BH, Taylor PR, Schatzkin A (2006). Diet and sex hormones in boys: findings from the dietary intervention study in children. J Clin Endocrinol Metab.

[B51] Campbell BC, Gillett-Netting R, Meloy M (2004). Timing of reproductive maturation in rural versus urban Tonga boys, Zambia. Ann Hum Biol.

[B52] Hauspie RC, Vercauteren M, Susanne C (1997). Secular changes in growth and maturation: an update. Acta Paediatr Suppl.

[B53] Ovesen L (2006). Adolescence: a critical period for long-term tracking of risk for coronary heart disease?. Ann Nutr Metab.

[B54] Arslanian S, Suprasongsin C (1997). Testosterone treatment in adolescents with delayed puberty: changes in body composition, protein, fat, and glucose metabolism. J Clin Endocrinol Metab.

[B55] Saad RJ, Keenan BS, Danadian K, Lewy VD, Arslanian SA (2001). Dihydrotestosterone treatment in adolescents with delayed puberty: does it explain insulin resistance of puberty?. J Clin Endocrinol Metab.

[B56] Wickman S, Saukkonen T, Dunkel L (2002). The role of sex steroids in the regulation of insulin sensitivity and serum lipid concentrations during male puberty: a prospective study with a P450-aromatase inhibitor. Eur J Endocrinol.

[B57] Hero M, Ankarberg-Lindgren C, Taskinen MR, Dunkel L (2006). Blockade of oestrogen biosynthesis in peripubertal boys: effects on lipid metabolism, insulin sensitivity, and body composition. Eur J Endocrinol.

[B58] Moran A, Jacobs DR, Steinberger J, Cohen P, Hong CP, Prineas R, Sinaiko AR (2002). Association between the insulin resistance of puberty and the insulin-like growth factor-I/growth hormone axis. J Clin Endocrinol Metab.

[B59] Goran MI, Gower BA (2001). Longitudinal study on pubertal insulin resistance. Diabetes.

